# Impact of smoking cessation, coffee and bread consumption on the intestinal microbial composition among Saudis: A cross-sectional study

**DOI:** 10.1371/journal.pone.0230895

**Published:** 2020-04-29

**Authors:** Steve Harakeh, Emmanouil Angelakis, Timokratis Karamitros, Dipankar Bachar, Suhad Bahijri, Ghada Ajabnoor, Sulaiman M. Alfadul, Suha A. Farraj, Turki Al Amri, Ahmed Al-Hejin, Abdalla Ahmed, Ahmed A. Mirza, Raoult Didier, Esam I. Azhar

**Affiliations:** 1 Special Infectious Agents Unit, King Fahd Medical Research Center, King Abdulaziz University, Jeddah, Saudi Arabia; 2 Aix Marseille Université, IRD, APHM, VITROME, IHU-Méditerranée Infection, Marseille, France; 3 Laboratory of Medical Microbiology, Hellenic Pasteur Institute, Athens, Greece; 4 Unit of Bioinformatics and Applied Genomics, Hellenic Pasteur Institute, Athens, Greece; 5 Clinical Biochemistry Department, College of Medicine, Nutrition unit, King Fahd Medical Research Center, King Abdulaziz University, Jeddah, Saudi Arabia; 6 King Abdulaziz city for Science and Technology, Riyadh, Saudi Arabia; 7 Family and Community Medicine department, Faculty of Medicine-Rabigh Branch, King Abdulaziz University, Jeddah, Saudi Arabia; 8 Biological Sciences Department, Faculty of Science, King Abdulaziz University, Jeddah, Saudi Arabia; 9 Department of Medical Microbiology, Faculty of Medicine, Umm Al-Qura University, Makkah, Saudi Arabia; 10 Department of Laboratory Medical Technology, Faculty of Applied Medical Sciences, King Abdulaziz University, Jeddah, Saudi Arabia; 11 Aix Marseille Université, IRD, APHM, MEPHI, IHU-Méditerranée Infection, Marseille, France; Wageningen Universiteit, NETHERLANDS

## Abstract

The gut microbiota is often affected by the dietary and lifestyle habits of the host, resulting in a better efficacy that favors energy harvesting from the consumed food. Our objective was to characterize the composition of gut microbiota in adult Saudis and investigate possible association with lifestyle and dietary practices. Feces from 104 Saudi volunteers (48% males) were tested for microbiota by sequencing the V3-V4 region of bacterial 16S ribosomal RNA (rRNA). For all participants, data were collected related to their lifestyle habits and dietary practices. The relative abundance (RA) of Fusobacteria was significantly higher in normal weight Saudis (*P * = 0.005, false discovery rate–FDR = 0.014). Individuals who consumed more coffee presented marginally significant more RA of Fusobacteria (*P * = 0.02, FDR = 0.20) in their gut microbiota compared to those reporting low or no coffee intake, but the RA of Fusobacteria was significantly higher in smokers compared to non-smokers (*P * = 0.009, FDR = 0.027). The RA of Fusobacteria was also significantly higher in those reporting daily consumption of bread (*P* = 0.005, FDR = 0.015). At the species level, the gut microbiota of people who consumed coffee was dominated by *Bacteroides thetaiotaomicron* followed by *Phascolarctobacterium faecium* and *Eubacterium rectale*. Similarly, the gut microbiota of smokers was also enriched by *B*. *thetaiotaomicron* and *Lactobacillus amylovorus*. Smoking cessation, bread and coffee consumption induce changes in the intestinal microbial composition of Saudis. This indicates the significance of diet and lifestyle practices in the determination of the composition of the gut microbiota, which could possibly lead later to changes in metabolic profile and weight.

## Introduction

The gastrointestinal microbiome establishes a stable symbiotic, mutually beneficial relationship with the host. However, it is affected by age, drugs and diet among other factors [1, 2]. Dietary and lifestyle habits positively alter the composition of gut microbiota to harvest energy from consumed food [3, 4]. Recent studies revealed that coffee has antibiotic effects and its consumption can modify the gut microbiota’s composition [5]. Similarly, smoking modifies the gut microbiota causing an increase in Firmicutes and Actinobacteria population and leads to a decrease in microbial diversity [6]. Moreover, the type of food intake by the human host also influences the gut microbiota composition and diversity [1, 2]. The gut microbiota is important to the process of harvesting, storing and expending energy attained from the diet. It has been reported that the microbiota of the duodenal aspirates from obese people was able to modulate fatty acid and sucrose breakdown pathways, likely due to dietary imbalance and help the host in harvesting energy and lead to increasing adiposity [7].

Saudi Arabia has rapidly developed economically and socially in the past decades with an impact on the lifestyle of its citizens. Such a rapid lifestyle change has influenced the dietary consumption habits of the whole population and resulted in a trend towards intake of high energy and processed fats with high levels of fat, salt and sugar [8]. As a result, obesity has appeared as an endemic disorder that is quickly emerging as a major problem in Saudi Arabia. According to a local study, around 70% of adult Saudis are either overweight or obese [9].

The body fat percentage among Saudi children and teenagers is increasing and leading to an emergence of obesity [10]. To date, there are few studies that have characterized the gut microbiota composition among Saudis [11]. The objective of this study was to use high-throughput 16S ribosomal RNA (rRNA) gene amplicon sequencing to characterize the gut microbiota of a Saudi population and determine if smoking, coffee and bread consumption had an impact on their microbiome.

## Methods

### Subject selection criteria

#### Subjects and study design

This cross-sectional study was conducted between January 2015 and December 2015 on healthy adults of both genders, aged 18–55 years and of different body mass index (BMI), recruited from the student population and others attending King Abdulaziz University Medical campus, as well as members of their families and friends. Exclusion criteria included: history of colon cancer, inflammatory bowel disease, acute or chronic diarrhea in the previous 8 weeks and treatment with antibiotics in the 2 months prior to fecal sampling, and intake of medication or supplements. The study was approved by the Ethics Committee of human Research at King Abdulaziz University under agreement number 014-CEGMR-2-ETH-P. All participants were asked to sign a written informed consent after being informed about the purpose of the study and ensured about confidentiality of the data. They were then requested to fill out a questionnaire covering their socio-demographic information, medical history and lifestyle practices. In addition, a structured food frequency questionnaire (FFQ) was administered to evaluate their dietary practices, and weight and height measurements were taken using standardized techniques. The used questionnaire was previously described partially or fully and used in other manuscripts [12–15]. Weight and height were used to calculate body mass index (BMI = kg m^-2^) and the WHO criteria [16] were used to classify participants as underweight, normal, overweight and obese. Weight categories were defined according to BMI as follows: normal 20–25 kg m^−2^, underweight 18–20 kg m^−2^, overweight 25–30 kg m^−2^, and obese >30 kg m^−2^. Stool samples were collected in aseptic conditions with clean, dry screw-top containers and immediately stored at -20 °C.

#### Extraction of DNA from stool samples and 16S rRNA sequencing using MiSeq technology

All participants’ stool samples were extracted using a deglycosylation protocol as follows: 250 μL of each sample was placed in a 2 mL tube containing a mixture of acid-washed glass beads (Sigma, Aldrich) and with two or three 0.5 mm glass beads. Mechanical lysis was performed by bead-beating the mixture using a Fast Prep BIO 101 apparatus (Qbiogene, Strasbourg, France) at maximum speed (6.5) for 3×30 seconds. The supernatant was centrifuged at 12,000 rpm for 10 min and the pellet retained. A mixture containing 2 μL of 10×glycoprotein denaturing buffer EndoHf (New England Biolabs) and 17 μL of H_2_O was added and heated at 100 °C for 10 minutes. Deglycosylation was performed adding a mixture of 2 μL of 10×G5 reaction buffer (ref B1702 New England Biolabs), 2 μL of EndoHf (New England Biolabs), 2 μL of cellulase (Sigma) and 16 μL of H_2_O. The preparation was then incubated overnight at 37 °C. Finally, DNA was extracted using the NucleoSpin^®^ Tissue Mini Kit (Macherey Nagel, Hoerdt, France) according to a previously described protocol [17]. The quantity, purity, integrity and size of DNA and its amenability to PCR amplification were assessed. The concentration of each DNA extraction was measured by a Qubit assay with the high sensitivity kit (Life technologies, Carlsbad, CA, USA) according to the Nextera XT DNA sample prep kit (Illumina) and diluted to 1 ng aliquots of each metagenome for paired end sequencing analysis. DNA extracts were dispensed into 10- to 20-μL single-use aliquots and frozen at -20 °C to avoid repeat freeze-thaw cycles prior to downstream analyses. Samples were then sequenced targeting the V3–V4 regions of the 16S rRNA gene using MiSeq technology as previously described [18, 19].

#### Data processing: Filtering the reads, dereplication and clustering

Paired end fastq files were assembled using FLASH [20]. A total of 7518258 joined reads were filtered and then analyzed in QIIME by choosing chimera slayer for removing chimera and Uclust [16, 20] for Operational Taxonomic Units (OTU) extraction as described previously [18, 19]. All reads were clustered with a threshold of 97% identity to obtain OTU. Extracted OTUs were blasted against SILVA123 SSU database [21] of release and taxonomy were assigned to a species if they matched one with at least 97% identity, as previously described [22, 23]. Briefly, for each OTU, representative sequences were extracted and were searched against the reference database. For each unique representative sequence, we extracted the best matches from the reference database and sorted them by decreasing percentage of similarity rounded to the nearest integer. We used the reference sequences with >97% similarity (or the highest available) for taxonomic assignments into species. When multiple matches with the same percentage of similarity were present, the taxonomy of each rank was obtained by consensus [16, 24]. OTU not assigned to any species were considered "unidentified". As several OTUs matched identical species, the total number of identified species and the number of unidentified OTU was expected to be smaller than the total number of OTUs.

#### Statistical analysis

A non-parametric Kruskal-Wallis test was used with an adjustment for multiple comparisons using the *post hoc* Benjamini-Hochberg correction from the OMICS package in XLSTAT V.2016.02 (Addinsoft, Paris, France). After normalization, an exploratory Pearson principal component analysis was first performed using the relative abundance at the bacterial phyla and general level as an active variable and age, sex, and coffee consumption, smoking and bread consumption as supplementary elements using XLSTAT v2014.3.07 (Addinsoft). Statistical analyses were performed using GraphPad Prism, V 5.0 (La Jolla, California, USA). The Fisher’s exact test was used for dichotomous variables, the Mann-Whitney test for continuous variables and the Spearman test for correlations. Principal coordinate analysis (PCoA) was obtained using the weighted unifrac distance after data rarefaction at the depth of 50,000 reads per sample, and the Adonis test was performed in QIIME [20, 25]. Linear discriminant analysis effect size (LEfSe) was performed on relative abundance at the genus and species levels using parameters previously recommended, including per-sample normalization of the sum of the values to 1 M (http://huttenhower.sph.harvard.edu/galaxy/) [23].

## Results

We tested 104 volunteers (48% males) with median age ± interquartile range (IR) was 24 ± 7.7 (Table 1).

**Table 1:** Description of the volunteers.

		Number of volunteers (%)	Median age ± Interquartile range
**BMI**	Underweight	21 (20.2%)	**23.0 ± 10**
	Normal weight	31 (29.8%)	**23.0 ± 6**
	Overweight	28 (26.9%)	**25.0 ± 8**
	Obese	24 (23.1%)	**27.0 ± 7**
**Smokers**		19 (19.2%)	**28.0 ± 8**
**Coffee**	Daily consumption	5 (5.0%)	**20.0 ± 8**
	Often consumption	53 (52.5%)	**25.0 ± 7**
	No consumption	43 (42.5%)	**24.0 ± 9**
**Bread**	Daily consumption	67 (67.0%)	**24.0 ± 8**
	Often consumption	21 (21.0%)	**26.0 ± 8**
	No consumption	12 (12.0%)	**25.0 ± 6**

### Composition of gut microbiota and body mass index

The analysis of the high-quality trimmed reads revealed that the gut microbiota of the subjects contained sequences mostly belonging to Firmicutes, followed by Bacteroidetes. The relative abundance of Fusobacteria was significantly higher (87%) in normal weight subjects (*P* = 0.005, FDR = 0.014). Lentisphaerae was marginally significantly higher in the gut microbiota of obese subjects than in the gut microbiota of normal (*P* = 0.01, FDR = 0.11) and underweight subjects (*P* = 0.008, FDR = 0.022). Lentisphaerae were not detectable in the gut microbiota of overweight subjects. Euryarchaeota and Synergistetes were not detectable in the gut microbiota of obese individuals. PCoA of the overall composition of the genera communities among the groups did not reveal differences among the microbiomes of underweight, normal weight, overweight and obese individuals (S1 Fig). The microbial richness estimated by the Shannon index did not reveal differences among the microbiomes of underweight, normal weight, overweight and obese individuals.

### Composition of gut microbiota and coffee consumption

**Fig 1:**
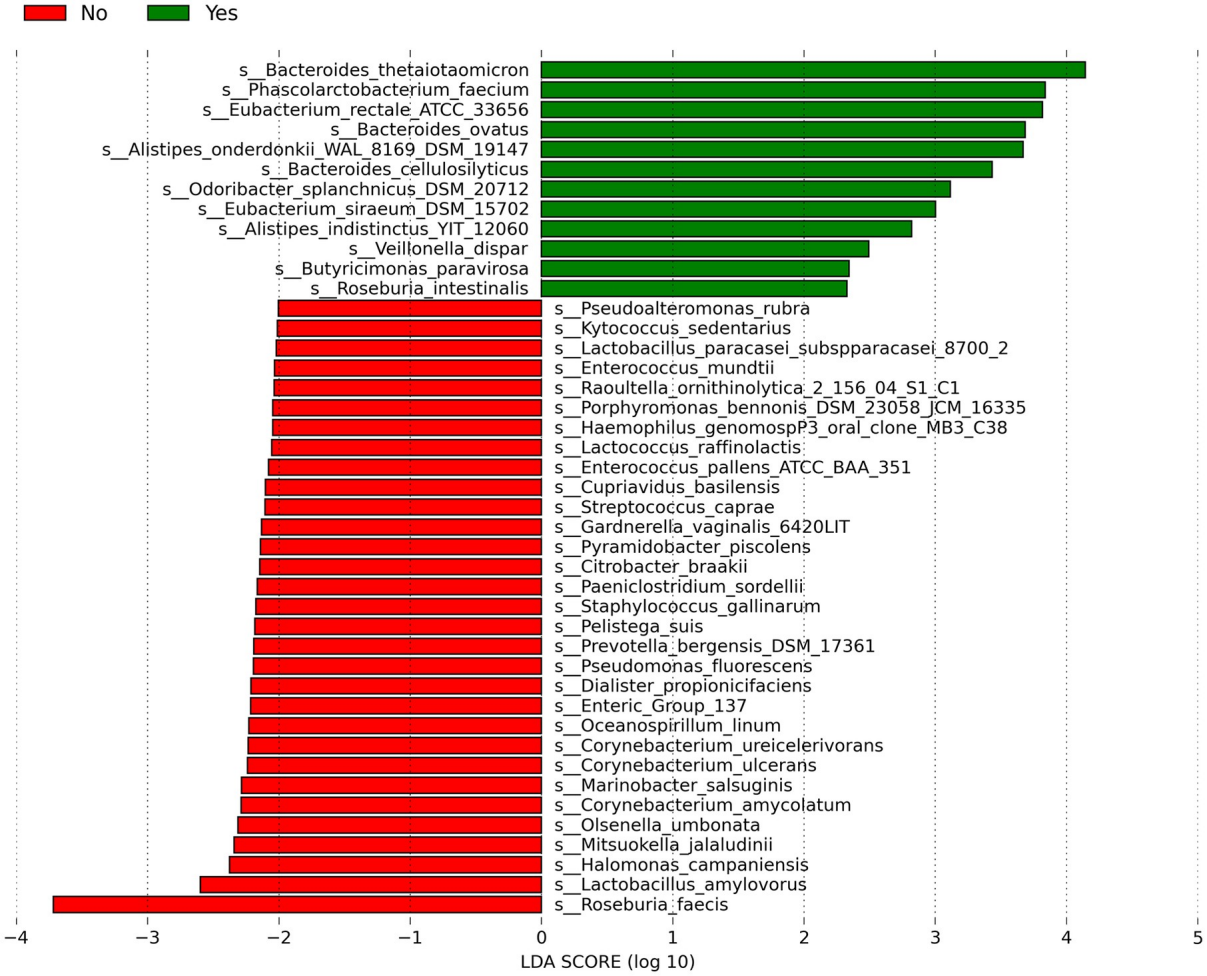
Linear Discriminant Analysis (LDA) scores of differentially abundant species among individuals who consume coffee (green) or not (red). The LDA scores represent the effect size of each abundant species. Species enriched in each group with an LDA score >2 are considered.

We found that coffee consumption influenced the gut microbiome of the subjects. Indeed, the gut microbiota of subjects who consumed coffee daily presented a significantly higher relative abundance of Synergistetes when compared to those who consumed coffee often (*P* = 0.01, FDR = 0.10) or not at all (*P* = 0.01, FDR = 0.08). Individuals who often consumed coffee presented marginally significantly more relative abundance of Fusobacteria (86%) than those who consumed coffee daily or not at all (*P* = 0.02, FDR = 0.20). Individuals who often consumed coffee daily or often had significantly more Tenericutes and Euryarchaeota than individuals who did not consume coffee. Moreover, PCoA of the overall composition of the genera communities between the groups did not reveal differences among the microbiomes of individuals concerning coffee consumption (S1 Fig). The microbial richness did not reveal differences among the microbiomes of subjects who consumed coffee daily, often or not at all (S2 Fig). *Bifido bacterium* spp relative abundance did not differ among individuals who consumed coffee or not (*P* = 0.6, FDR >0.2). However, we found that the gut microbiota of subjects who consumed coffee daily was enriched by *Eubacterium rectale* whereas the microbiota of those who did not consume coffee was enriched by *Marinomona sarctica*. The gut microbiota of individuals who consumed coffee often was enriched by *Bacteroides thetaiotaomicron* (S3 Fig). Overall, the gut microbiota of subjects who consumed coffee was dominated by *B*. *thetaiotaomicron* followed by *Phascolarcto bacterium faecium* and *E*. *rectale*, whereas the gut microbiota of subjects who did not consume coffee was dominated by *Roseburia faecis* (Fig 1).

### Composition of gut microbiota and bread consumption

The relative abundance of Fusobacteria (88%) was significantly higher in the gut microbiota of subjects who daily consumed bread (*P* = 0.005, FDR = 0.015). Fusobacteria were not detectable in the gut microbiota of subjects who did not consume bread. Similarly, the relative abundance of Synergistetes and Lentisphaerae were significantly higher in the gut microbiota of subjects who often consumed bread (*P* = 0.009, FDR = 0.028 and *P* = 0.004, FDR = 0.011 respectively). Synergistetes, Cyanobacteria, Euryarchaeota and Lentisphaerae were not detectable in the gut microbiota of subjects who did not consume bread. PCoA of the overall composition of the genera communities among the groups did not reveal differences among subjects concerning bread consumption (S1 Fig). Moreover, bread consumption did not affect the gut microbial richness (S2 Fig).

### Composition of gut microbiota in relation to smoking

**Fig 2:**
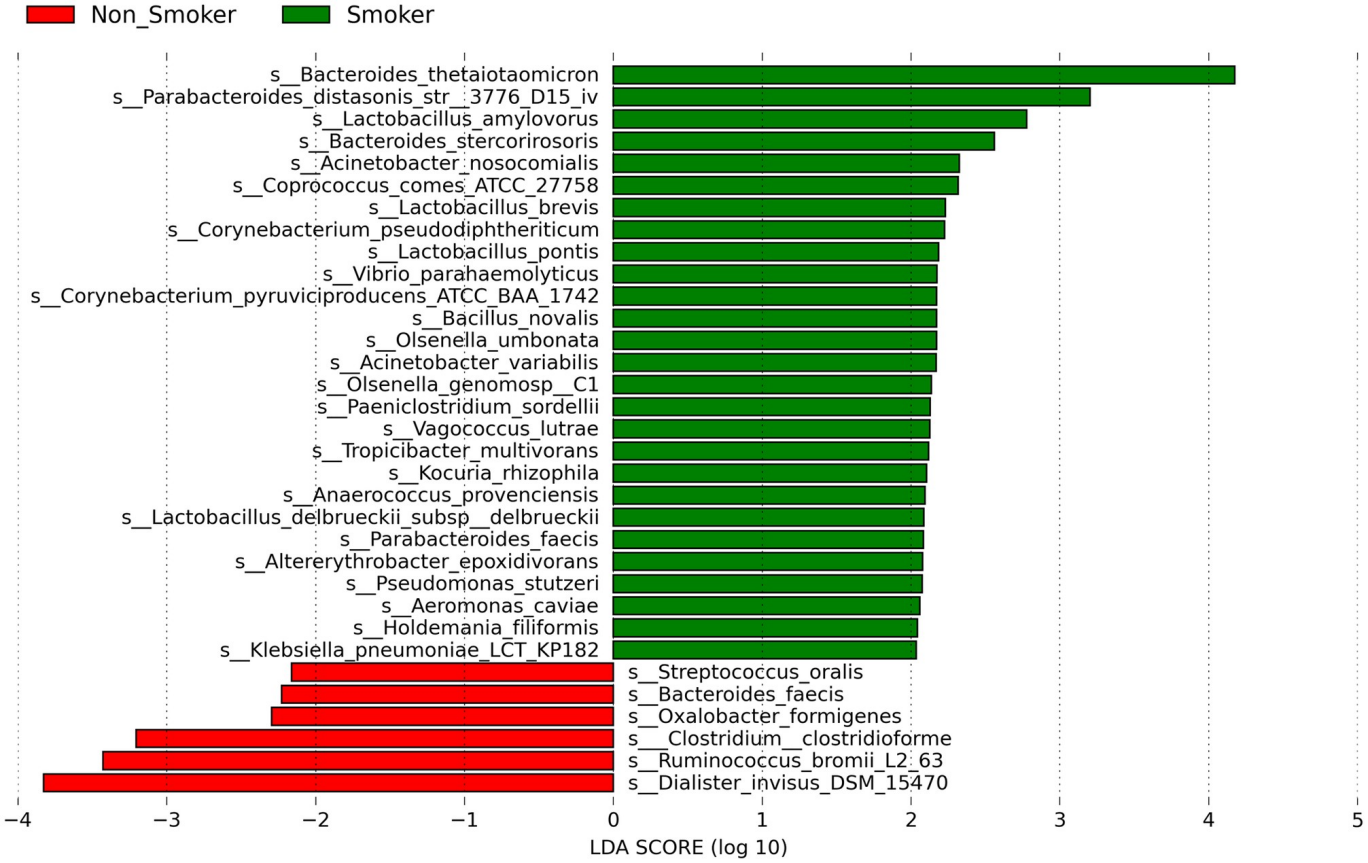
LDA scores of differentially abundant species according to smokers (green) and individuals who do not smoke (red). The LDA scores represent the effect size of each abundant species. Species enriched in each group with an LDA score >2 are considered.

**Fig 3:**
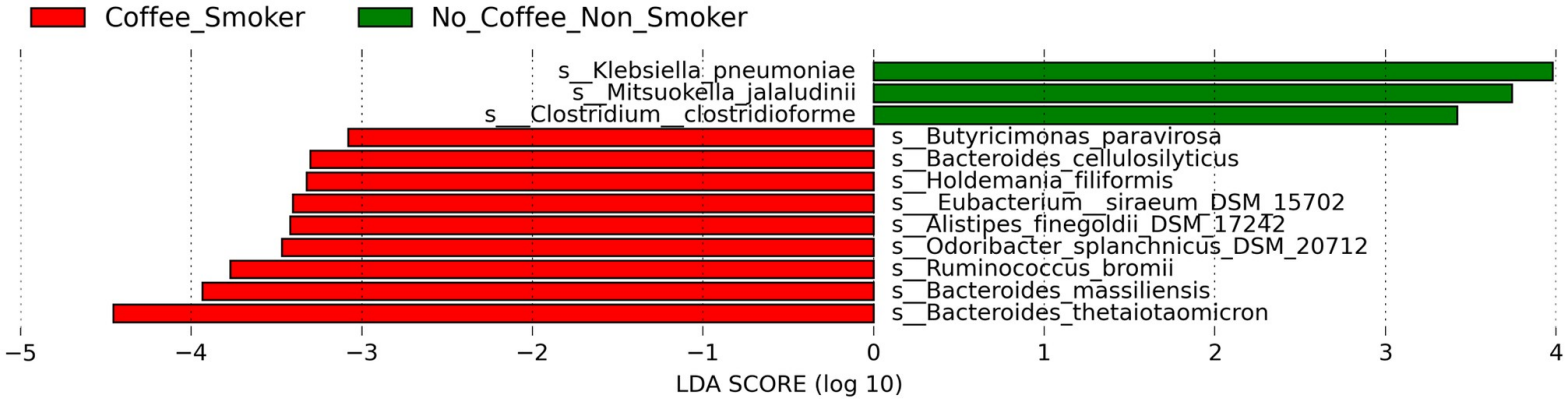
LDA scores of differentially abundant species according to individuals who consumed coffee and were smokers (red) and individuals who do not smoke and consume coffee (green). The LDA scores represent the effect size of each abundant species. Species enriched in each group with an LDA score >2 are considered.

The analysis of the high-quality trimmed reads revealed that the gut microbiota of subjects who were smokers contained sequences mostly belonging to Bacteroidetes. Moreover, the gut microbiota of the non-smokers had a significantly higher relative abundance of Fusobacteria (83%) (*P* = 0.009, FDR = 0.027) and Tenericutes (*P* = 0.008, FDR = 0.018) than the gut microbiota of smokers. Synergistetes, Lentisphaerae and Euryarchaeota were not detectable in the gut microbiota of the non-smokers. PCoA of the overall composition of the genera communities between the groups did not reveal differences between the microbiomes of smokers and non-smokers (S1 Fig). Similarly, we did not find a difference in the microbial richness, between the gut microbiomes of smokers and non-smokers (S2 Fig). Analysis at the species level revealed that the gut microbiota of smokers was also enriched by *B*. *thetaiotaomicron* and *Lactobacillus amylovorus*, whereas the gut microbiota of the individuals who did not smoke was enriched by *Dialister invisus* and *Ruminococcus bromii* (Fig 2). Finally, we found that the gut microbiota of subjects who were smokers and who consumed coffee were dominated by *B*. *thetaiotaomicron* followed by *Bacteroides massiliensis*, whereas the gut microbiota of subjects who did not smoke and consumed coffee by *Klebsiella pneumoniae* (Fig 3).

## Discussion

In this study, we compared the gut microbiomes of Saudis and we provide important information about the impact of different dietary habits on their gut microbiome. To the best of our knowledge, this is the second attempt to evaluate the statistical variability of the gut microbiota among Saudis. Moreover, this is the first study that involved female volunteers as Saudi Arabia has a conserved society and people are reluctant to volunteer for studies involving stool samples, particularly females. A limitation of our study was that we did not measure on our Saudi volunteers the waist circumference, visceral fat and the fat content (%), which are more relevant factors than BMI. Metagenomics studies are the preferential technique for the exploration of the gut microbiota diversity but have considerably low reproducibility due to the differences in sampling, sample conservation, DNA extraction protocol, sequencing method, and data analysis strategy [7, 11, 18, 26]. Although next generation sequencing (NGS) technology has also been used in several studies for the exploration of the gut microbiota to the species level [7, 11, 18], an unequivocal identification on the species level is not always possible due to the high similarities of the 16S rRNA gene sequencing in some bacterial species and due to the small sequence that is obtained that is roughly one third of the full 16S rRNA gene.

Previously, Bacteroidetes have been linked to weight gain [1]. The reasons for the changes of the complex gastrointestinal microbiome ecosystem due to obesity are still controversial. In our previous pilot study, we evaluated changes of the gut microbiota of Saudis and reported that Saudis had a significantly lower diversity in comparison to a group of French people. The difference may have been due to the food intake with less diversity among Saudis in comparison to the French [11]. Moreover, we found that obese Saudis possessed significantly more Firmicutes than normal weight Saudis [11]. However, we did not find differences in the gut microbiota population of Firmicutes or Bacteroidetes among individuals with different weight phenotype in this study. Contradictory results on the microbiome can be noted from different analyses conducted from the same laboratory [26, 27]. In our study we believe that these discrepancies were due to subject selection. In a recent systematic review based on animal experiments and clinical studies, it was found that a weight loss after a bariatric surgery was associated with an increase of Fusobacteria in the gut microbiota [28]. This may explain our findings that Fusobacteria were significantly higher in normal weight Saudis compared to the overweight or obese ones.

Diet plays an important role on the gut microbiomes and can influence the bacterial diversity from carnivore to omnivore to herbivore [29]. Indeed, there are various reports of the influence of dietary components, as well as lifestyle on gut microbiota in humans from different geographical regions [28–34].

A high-fiber diet has been associated with an enrichment of the gut microbiota [31], and gut microbiota differences were associated with different types of diet [32–34]. Previous reports have indicated that Western populations have lower microbial richness than non-Western populations [32]. In a previous study we found that the gut microbiota of Bedouins, a nomadic population in Saudi Arabia, eating a diet that is mostly based on vegetables, fruits, chicken, dairy products, fermented food and rice presented an increased biodiversity when compared to urban Saudis [18]. Even though bread consumption did not affect the gut microbial richness in our study, the relative abundance of Fusobacteria was significantly higher in the gut microbiota of subjects consuming bread on a daily basis, while they were not detectable in the gut microbiota of those who did not consume bread. Similarly, the relative abundance of Synergistetes and Lentisphaerae were significantly higher in the gut microbiota of subjects who often consumed bread but were not detectable in the gut microbiota of those who did not consume bread. Similar effects on microbial richness were reported in mice fed whole-wheat bread, and the relative abundance of microbial species also differed [35]. However, a recent human study reported absence of a significant species compositional change in the microbiota of persons fed either traditionally made sourdough leavened whole-grain bread or industrially made white bread for one week, even though some clinical parameters and the glycemic index of the subjects were affected [36]. The researchers proposed that the short period of the study was not enough to induce changes in the gut microbiota. However, altering the diet will quickly lead to the modulation of the gut microbiome, thus facilitating the adaptability to diverse human lifestyles diets [35, 36].

We also found that coffee consumption influences the gut microbiota of Saudis. Few studies have evaluated the relationship between coffee consumption and gut microbiota [5]. Gniechwitz *et al*. found that the fecal microbiota did not change after coffee consumption [37]. Similarly, healthy adults who consumed three cups of coffee daily for three weeks did not present important modifications to their gut microbiota [38]. In contrast, some individuals presented an increase in the population of *Bifidobacterium* spp in their gut microbiota [38]. In our studied individuals, we did not find that coffee consumption influenced the population of gut *Bifidobacterium* spp. However, we found that the gut microbiota of people who consumed coffee often was enriched by *E*. *rectale* and *B*. *thetaiotaomicron*. The same bacterial species also dominated in the gut microbiota of smokers. In a recent study, the fecal microbiota of individuals undergoing smoking was also enriched by *Clostridium coccoides*, *E*. *rectale*, and *Clostridium leptum* subgroup [6, 39]. In addition, a cross-sectional study using fluorescent *in situ* hybridization targeting selected bacterial groups reported that smokers suffering by Crohn’s disease presented higher Bacteroidetes–*Prevotella* comparing to nonsmoking patients [40]. It was previously found that the intestinal bacterial composition and diversity began to change from the fourth week after smoking cessation. Although our study was not designed to investigate potential causative mechanisms of the smoking cessation on the gut microbiota, smoking seems to affect bowel mucosa and mucin expression. Moreover, it is possible that the absorption of several toxic chemical compounds in cigarette smoke could change metabolism and alter the composition of gut microbiota. In addition, immune modification due to smoking may indirectly affect the composition of gut microbiota.

## Conclusion

We provide evidence that smoking cessation and coffee consumption induces changes in the intestinal microbial composition of Saudis. Food affects the gut microbiota of individuals and we proved that bread consumption modified that gut microbiota of Saudis. Most of these gut microbiota modifications affect the population of Fusobacteria, a phylum that is not a large member within human gut microbiota. We point out the importance of more intensive research in the future to understand the impact of Fusobacteria as also of diet, coffee and smoking cessation on the gut microbiota.

## Supporting information

S1 Fig Principle coordinate analysis (PCoA) of the overall composition of the genera communities among the groups. (TIF)

S2 Fig Gut microbiota Chao1 indexes according to a, coffee consumption; b, smoking; and c, bread consumption. (TIF)

S3 Fig LDA scores of differentially abundant species among individuals based on daily (red) or often (blue) coffee consumption and no coffee consumption (green). The LDA scores represent the effect size of each abundant species. Species enriched in each group with an LDA score >2 are considered.
(TIF)
