# Correction: Impact of smoking cessation, coffee and bread consumption on the intestinal microbial composition among Saudis: A cross-sectional study

**DOI:** 10.1371/journal.pone.0318869

**Published:** 2025-01-30

**Authors:** The PLOS One Editors

This Correction resolves the prior Expression of Concern for the linked article [1, 2].

Following the publication of the article and Expression of Concern [1, 2], PLOS investigated the concerns pertaining to the reported ethical oversight and the article’s adherence to PLOS Human Subjects Research policies.

Specifically, PLOS found that the ethics approval number #014-CEGMR-2-ETH-P was also reported in multiple other publications [3–19], despite apparent differences in the study objectives, periods during which the data were collected, study locations, study populations, sample sizes, sampling methodologies and ethics approval restrictions to data collection described in these studies.

Co-author SB responded stating that the incorrect ethics approval was reported during the preparation of the manuscript. They provided the study protocol submitted for ethics review and the ethics approval document #150–14, issued by the Research Ethics Committee of the King Abdulaziz University, Saudi Arabia, for a study titled “*Microbiota biomarkers in obese patients in Saudi Arabia*”. The approval was issued on 14 May 2014 and was valid for one year. The PLOS article [1] reports that data were collected between January 2015-December 2015. The corresponding author SH stated that the dates for data collection were reported incorrectly in [1], and co-author SB noted that the samples described in this article [1] were collected between May 20^th^ and September 30^th^ 2014.

A representative of the Research Ethics Committee of the King Abdulaziz University confirmed that the study described in this article [1] was approved by the institute’s ethics committee under approval #150–14. (Note: Ethics approval #150–14 was also cited in [20].)

In light of the information and documentation provided by the authors and the Research Ethics Committee of the King Abdulaziz University, the Subjects and Study design subsection of the Methods section is updated to:

This cross-sectional study was conducted between May 20^th^ 2014 and September 30^th^ 2014 on healthy adults of both genders, aged 18–55 years and of different body mass index (BMI), recruited from the student population and others attending King Abdulaziz University Medical campus, as well as members of their families and friends. Exclusion criteria included: history of colon cancer, inflammatory bowel disease, acute or chronic diarrhea in the previous 8 weeks and treatment with antibiotics in the 2 months prior to fecal sampling, and intake of medication or supplements. The study was approved by the Research Ethics Committee of the King Abdulaziz University on May 14^th^ 2014 (reference #150–14). All participants were asked to sign a written informed consent after being informed about the purpose of the study and ensured about confidentiality of the data. They were then requested to fill out a questionnaire covering their socio-demographic information, medical history and lifestyle practices. In addition, a structured food frequency questionnaire (FFQ) was administered to evaluate their dietary practices, and weight and height measurements were taken using standardized techniques. The used questionnaire was previously described partially or fully and used in other manuscripts [12–15]. Weight and height were used to calculate body mass index (BMI = kg m-2) and the WHO criteria [16] were used to classify participants as underweight, normal, overweight and obese. Weight categories were defined according to BMI as follows: normal 20–25 kg m−2, underweight 18–20 kg m−2, overweight 25–30 kg m−2, and obese >30 kg m−2. Stool samples were collected in aseptic conditions with clean, dry screw-top containers and immediately stored at -20 °C.

With this update, the *PLOS One* Editors consider the ethics approval concerns resolved. This Correction supersedes the prior Expression of Concern [2].
