# Supplementary figures and images for: Impact of smoking cessation, coffee and bread consumption on the intestinal microbial composition among Saudis: A cross-sectional study
Source: PLoS One. 2020 Apr 29;15(4):e0230895. doi: 10.1371/journal.pone.0230895 (PMC7190147; doi:10.1371/journal.pone.0230895)

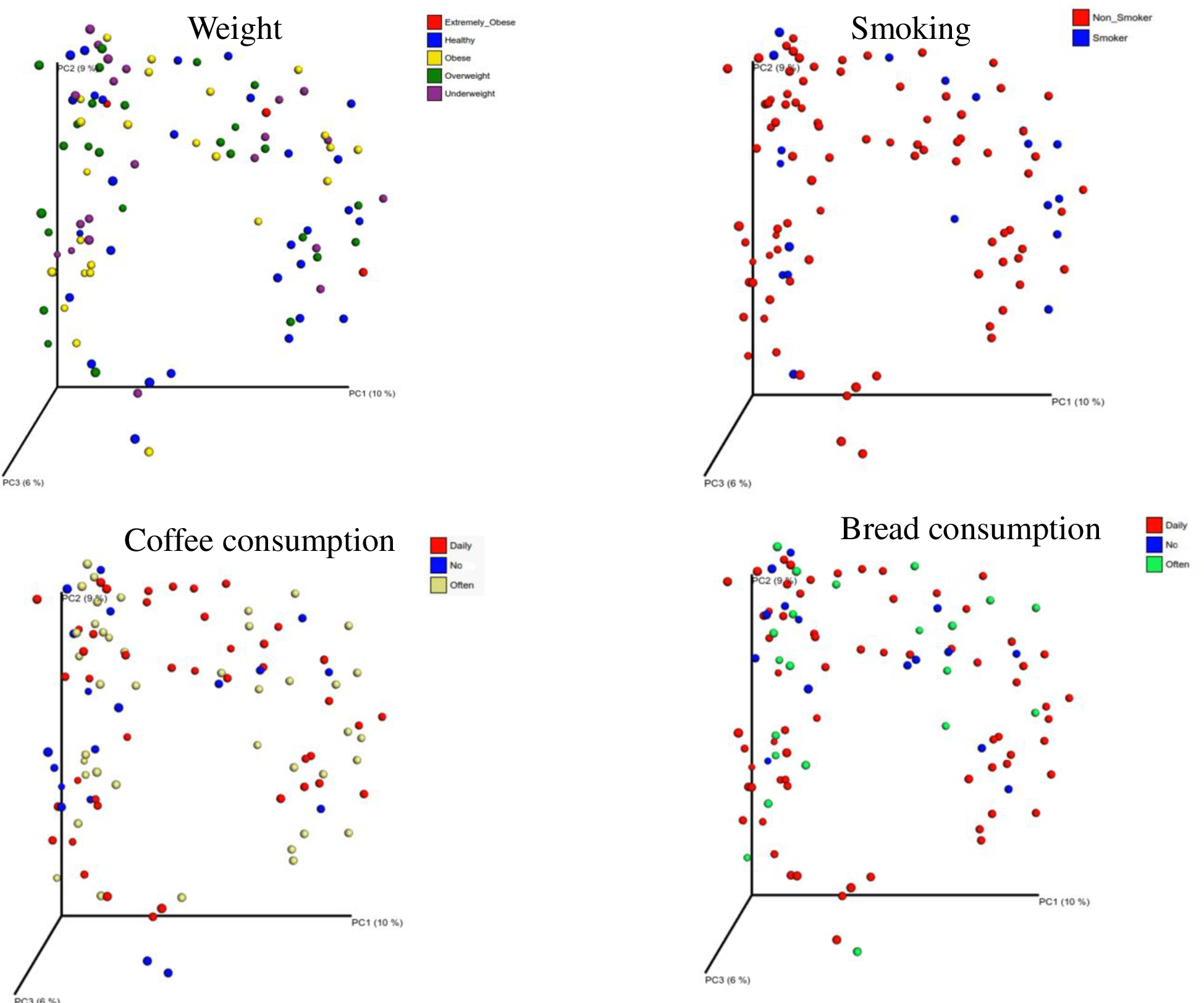

Supplement: S1 Fig — (TIF) [file pone.0230895.s001.tif]

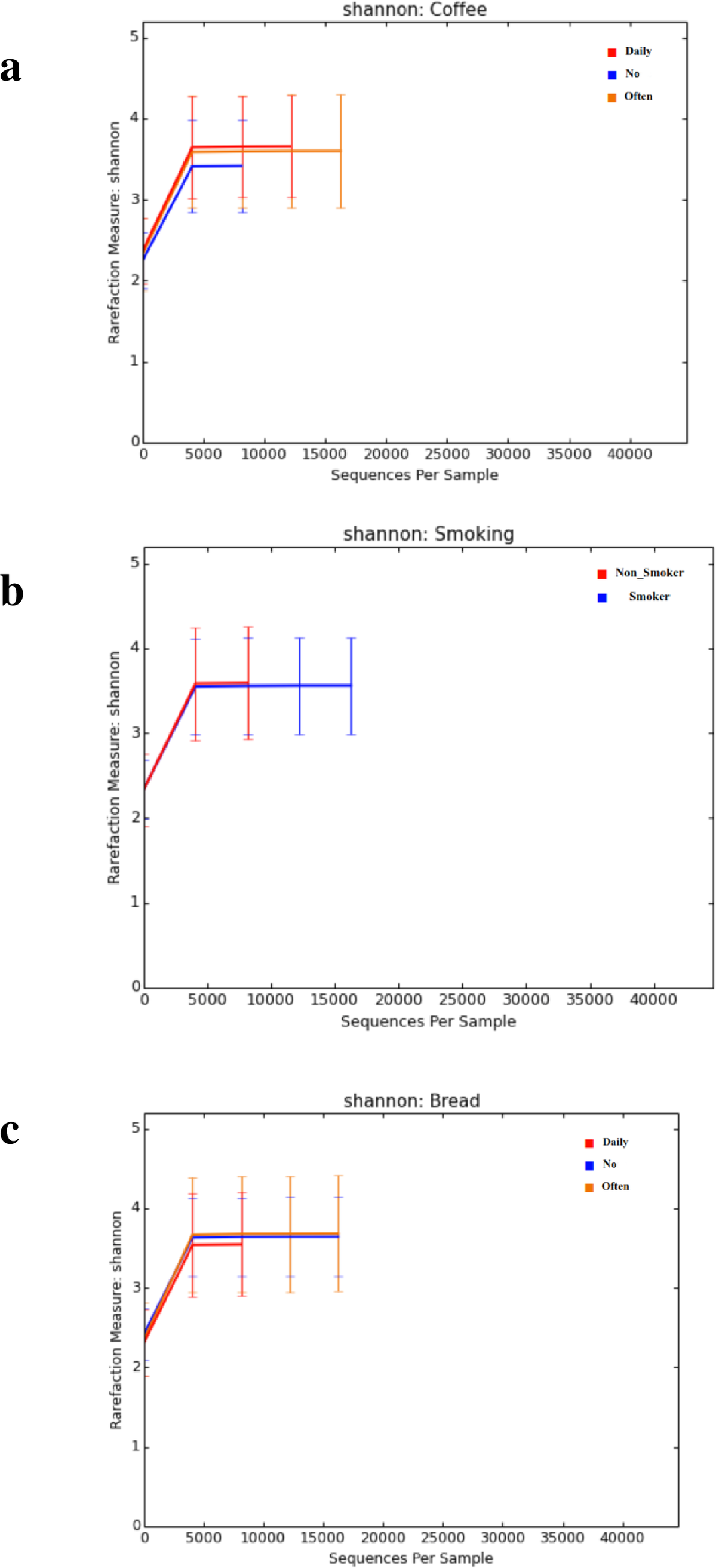

Supplement: S2 Fig — (TIF) [file pone.0230895.s002.tif]

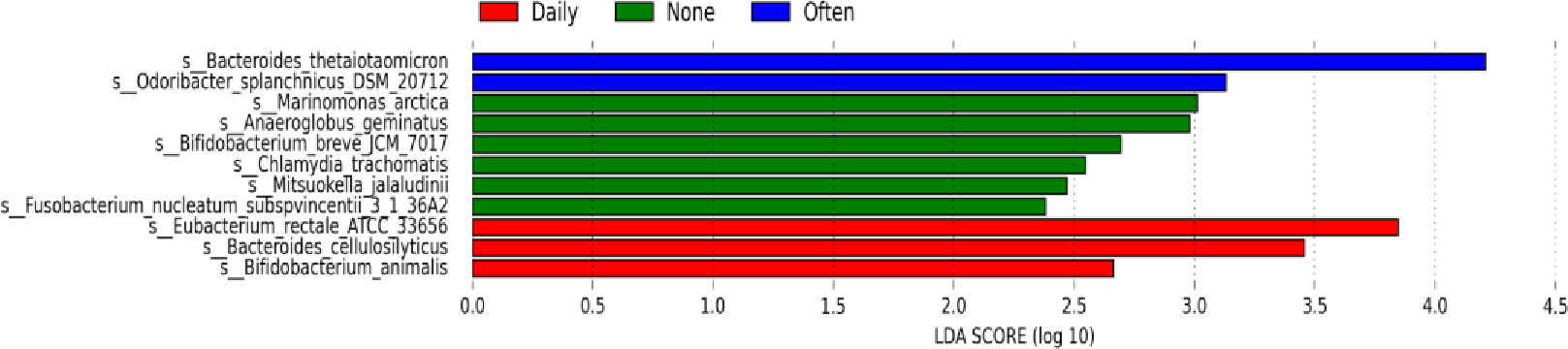

Supplement: S3 Fig — The LDA scores represent the effect size of each abundant species. Species enriched in each group with an LDA score >2 are considered. (TIF) [file pone.0230895.s003.tif]
